# Examining How Adults With Diabetes Use Technologies to Support Diabetes Self-Management: Mixed Methods Study

**DOI:** 10.2196/64505

**Published:** 2025-03-25

**Authors:** Timothy Bober, Sophia Garvin, Jodi Krall, Margaret Zupa, Carissa Low, Ann-Marie Rosland

**Affiliations:** 1 Caring for Complex Chronic Conditions Research Center Department of Medicine, Division of General Internal Medicine University of Pittsburgh Pittsburgh, PA United States; 2 VA Pittsburgh Center for Health Equity Research and Promotion Pittsburgh, PA United States; 3 University of Pittsburgh Medical Center Pittsburgh, PA United States; 4 Division of Endocrinology and Metabolism Department of Medicine University of Pittsburgh Pittsburgh, PA United States; 5 Mobile Sensing + Health Institute (MoSHI) Department of Medicine, Division of Hematology/Oncology University of Pittsburgh Pittsburgh, PA United States

**Keywords:** diabetes, self-management, mobile health, health technology, continuous glucose monitors, digital health literacy

## Abstract

**Background:**

Technologies such as mobile apps, continuous glucose monitors (CGMs), and activity trackers are available to support adults with diabetes, but it is not clear how they are used together for diabetes self-management.

**Objective:**

This study aims to understand how adults with diabetes with differing clinical profiles and digital health literacy levels integrate data from multiple behavior tracking technologies for diabetes self-management.

**Methods:**

Adults with type 1 or 2 diabetes who used ≥1 diabetes medications responded to a web-based survey about health app and activity tracker use in 6 categories: blood glucose level, diet, exercise and activity, weight, sleep, and stress. Digital health literacy was assessed using the Digital Health Care Literacy Scale, and general health literacy was assessed using the Brief Health Literacy Screen. We analyzed descriptive statistics among respondents and compared health technology use using independent 2-tailed *t* tests for continuous variables, chi-square for categorical variables, and Fisher exact tests for digital health literacy levels. Semistructured interviews examined how these technologies were and could be used to support daily diabetes self-management. We summarized interview themes using content analysis.

**Results:**

Of the 61 survey respondents, 21 (34%) were Black, 23 (38%) were female, and 29 (48%) were aged ≥45 years; moreover, 44 (72%) had type 2 diabetes, 36 (59%) used insulin, and 34 (56%) currently or previously used a CGM. Respondents had high levels of digital and general health literacy: 87% (46/53) used at least 1 health app, 59% (36/61) had used an activity tracker, and 62% (33/53) used apps to track ≥1 health behaviors. CGM users and nonusers used non-CGM health apps at similar rates (16/28, 57% vs 12/20, 60%; *P*=.84). Activity tracker use was also similar between CGM users and nonusers (20/33, 61% vs 14/22, 64%; *P*=.82). Respondents reported sharing self-monitor data with health care providers at similar rates across age groups (17/32, 53% for those aged 18-44 y vs 16/29, 55% for those aged 45-70 y; *P*=.87). Combined activity tracker and health app use was higher among those with higher Digital Health Care Literacy Scale scores, but this difference was not statistically significant (*P*=.09). Interviewees (18/61, 30%) described using blood glucose level tracking apps to personalize dietary choices but less frequently used data from apps or activity trackers to meet other self-management goals. Interviewees desired data that were passively collected, easily integrated across data sources, visually presented, and tailorable to self-management priorities.

**Conclusions:**

Adults with diabetes commonly used apps and activity trackers, often alongside CGMs, to track multiple behaviors that impact diabetes self-management but found it challenging to link tracked behaviors to glycemic and diabetes self-management goals. The findings indicate that there are untapped opportunities to integrate data from apps and activity trackers to support patient-centered diabetes self-management.

## Introduction

### Background

Adults with diabetes can significantly lower their risk of diabetes complications such as nerve damage, kidney failure, blindness, myocardial infarction, and stroke by maintaining healthy daily diabetes self-management behaviors [1-3]. Diabetes self-management behaviors span multiple domains, including taking medications on a consistent schedule, engaging in regular physical activity, maintaining a healthy diet, self-monitoring blood glucose levels and blood pressure, practicing good sleep hygiene, and managing stress [4,5]. Adults with diabetes navigate these self-management behaviors to make daily decisions unique to their treatment regimens—such as adjusting medication doses and food intake after exercising. Successfully changing and sticking with healthy self-management routines is challenging for many people [6,7]. These demanding tasks can also lead to overwhelming diabetes distress, which can result in less motivation to stick to healthy regimens as well as higher blood glucose levels [8].

Technologies such as mobile apps, wearable activity trackers, and wearable continuous glucose monitors (CGMs) are available to support adults with diabetes; however, little is known about how adults with diabetes use CGMs in combination with mobile apps and activity trackers for other diabetes self-management domains. With 1 in 5 Americans reporting that they use a smartwatch or fitness tracker [9], there are increasing opportunities for adults with diabetes to use these devices for diabetes management, as evidenced by their incorporation into diabetes treatment guidelines [10]. While CGM use has been linked to lower glycated hemoglobin levels among people with type 2 diabetes who use insulin [11,12], available evidence is not clear on whether using CGMs enables patients to improve medication taking or other diabetes self-management behaviors [13,14]. There is mixed evidence on whether using activity trackers results in lower blood glucose levels for adults with diabetes [15-17]. It remains unclear whether and how adults with diabetes connect data from these apps with information from their CGMs to guide daily behavioral routines. Other domains, including stress and sleep—increased stress has been linked to higher blood glucose levels among adults with type 1 and 2 diabetes [18]—are included as behaviors that are important to address in treatment guidelines [5], but we do not know whether adults with diabetes use technologies to track these behaviors and link them with their diabetes information.

### Objectives

In this mixed methods study, we aimed to assess how adults with diabetes use and combine blood glucose level and self-management behavior tracking technologies to inform their day-to-day diabetes self-management and reach their personal health goals. Our research question focuses on understanding how adults with diabetes with differing clinical profiles and digital health literacy use and integrate data from multiple behavior tracking technologies for diabetes self-management.

## Methods

### Study Population

Individuals aged 18 to 75 years with diabetes (type 1 or 2) who were prescribed at least 1 diabetes medication were eligible for this study. The age cutoff for the study was set at 75 years because diabetes management goals change once individuals are beyond a certain age, including more liberal blood glucose level targets that emphasize safety. This could affect the appropriateness of certain technologies, such as CGMs, and how individuals use them.

The exclusion criteria included a diagnosis of gestational diabetes without another diabetes diagnosis, a diagnosis of schizophrenia or any other kind of “serious mental illness,” and diagnoses of “serious medical illnesses” (eg, cancer, chronic obstructive pulmonary disease, and end-stage kidney disease).

### Recruitment

Potentially eligible participants were either referred by health care providers (n=2) or recruited via a posting on the University of Pittsburgh Pitt+Me research registry, which was available from March to November 2023. The posting contained information about the purpose of the study; eligibility criteria; and what participation involved, including with regard to completing the survey on the internet and potentially being invited for an interview. The target audience included individuals diagnosed with diabetes who are currently prescribed at least 1 diabetes medication. Potential participants who had responded to the posting were contacted via telephone by a study team member to confirm eligibility and obtain verbal consent (refer to the *Ethical Considerations* subsection for details). The study team member then administered the survey over the telephone or via a web-based Qualtrics (Qualtrics International Inc) form. After completing the survey, potential interview participants were purposefully recruited to represent a variety of experiences with technology and diabetes-related factors such as diabetes type, diabetes medication use (insulin or oral), and CGM use (current or prior), as well as demographic factors, including age, sex, and racial identity and ethnicity.

### Closed-Ended Survey Questions

The web-based survey (Multimedia Appendix 1 [19,20]) included 46 questions about sociodemographic characteristics; functional challenges to using apps (vision or dexterity problems); and diabetes management, including blood glucose level monitoring patterns. The survey took approximately 10 (median 9.31, IQR 11.85-18.66) minutes to complete; included free-text, multiple-choice, and Likert-scale response options; and was developed internally by the research team through multiple versions. We assessed current or previous participant experience with 6 types of mobile apps for diabetes self-management behaviors that correspond to key self-management domains in American Diabetes Association diabetes management guidelines [5]: blood glucose level monitoring, diet, exercise, weight loss, sleep, and stress and mindfulness. While current and previous technology use represent different use patterns, we decided to group these patterns together because these data could be used to craft interventions that incorporate different domains that adults with diabetes have demonstrated interest in tracking. We also asked about current and former use of wearable blood glucose level and activity trackers. We used questions from previously validated surveys, including the Brief Health Literacy Screen [19,21] for general health literacy, which is scored on a scale ranging from 3 to 15, with scores of ≤9 reflecting marginal or inadequate health literacy [22,23]; and the Digital Health Care Literacy Scale (DHLS) [20] reflecting the ability to use mobile apps (scored from 0 to 12, with higher scores indicating higher digital health literacy).

### Interviews

Semistructured interviews were conducted using a secure videoconferencing platform (Zoom; Zoom Video Communications, Inc) from May to November 2023. All interviews were conducted by a study team member with a doctorate degree in nutritional sciences, subject matter expertise, and training and experience in conducting interviews. Another study team member who is a primary care physician and expert in health literacy also participated and asked follow-up questions ad hoc. A trained research assistant took notes during the interviews and wrote reflexive memos summarizing each interview afterward. The interview guide (Multimedia Appendix 2) included questions for participants about their experiences using health technology to track data and make diabetes-related behavior changes. The guide was developed by study team members, pretested with 3 adults (who were not included in the data analysis), and revised based on their responses. In the final guide, participants were first asked to describe how they tracked information during a typical day and their experiences with diabetes-related mobile apps (6 types: blood glucose level monitoring, diet, exercise, weight loss, sleep, and stress and mindfulness) and wearable blood glucose level and activity trackers. We then asked participants to describe how they learned to use these apps and trackers; what features helped them manage diabetes; what aspects of the tools or training could have been more helpful; whether they combined information if they used >1 tool; and why they stopped using the tool, if applicable. In addition, participants were asked whether they shared blood glucose level data with members of their health care team or others (eg, family members); and to describe these experiences and how, if at all, they could be enhanced. The interviews took 45 to 60 minutes to complete and were recorded and transcribed. Transcripts were reviewed for accuracy by the research assistant.

### Data Analysis

#### Survey

Descriptive statistics—frequency (percentage) for categorical variables and mean (SD) for continuous variables—were used to assess patient characteristics and summarize other data (eg, health technology use, general health literacy, and digital health literacy). Participant use of each of the 6 types of apps and 2 types of wearables was tallied by category. We then categorized participants as 0, 1, or >1 app and wearable used. Health technology use was compared among participant groups (eg, age and insulin use) using independent 2-tailed *t* tests for continuous variables and chi-square for categorical variables. Data are presented as frequency (percentage) or mean (SD), unless otherwise noted. To capture as many aspects of technology use among adults with diabetes as possible, we did not exclude participant responses if they did not complete all questions in a particular section. Data were analyzed using SPSS (version 28.0; IBM Corp) and Stata (version 18.0 for Mac; StataCorp LLC).

To analyze the association between health literacy and app use, we used a cutoff score of ≤9 as a marginal or inadequate score based on prior literature [22,23]. As there are no defined tiers for low, marginal, or high levels of digital health literacy in the DHLS, we created categories for this score, including ≤9 (marginal or inadequate digital health literacy) and ≥10 (adequate digital health literacy) based on the distribution of scores among the survey respondents. We tested the association between digital health literacy and total app use using Fisher exact tests with an assigned α of .05.

#### Interviews

We used content analysis to summarize themes by interview topic [24,25]. An initial inductive codebook was developed based on the anticipated categories of information that would be gathered during the interviews. Members of the research team separately coded 3 transcripts, compared results as a group, and then adjusted the codebook to include deductive emerging themes. After reaching sufficient agreement on the dually coded transcripts, single-user coding was applied to the remaining transcripts. Team members separately reviewed coded passages and potential themes, which were then presented and discussed as a group until consensus was reached. Analysis was conducted using NVivo software (version 14 for Mac and Windows; Lumivero).

### Ethical Considerations

This study was deemed exempt by the University of Pittsburgh Institutional Review Board (22120073-001).

For the survey consent process, a study team member contacted interested individuals and confirmed their eligibility. The team member then outlined the goals of the study, the processes for completing the survey, the possibility of being contacted for a postsurvey interview, the potential risks and benefits of participation, the processes for protecting confidentiality and data safety, and the option of declining participation at any time. These parameters were reviewed with interview participants, who were selected from the survey respondents, at the beginning of their interviews.

Study data—including survey responses and interview quotes—were deidentified, labeled with a study ID number, and stored on Health Insurance Portability and Accountability Act–compliant servers. All personally identifying information was removed from transcripts.

Participants received US $10 in compensation for completing the survey and US $30 in compensation for completing the interview.

## Results

### Survey

A total of 61 adults with diabetes completed the survey. Of the 61 respondents, 60 (98%) owned a smartphone, and 54 (94%) used web-based resources to look up health information. As shown in Table 1, of the 61 respondents, 21 (34%) were Black, and 23 (38%) were female. Approximately half (29/61, 48%) were aged 45 to 70 years, and most (50/61, 82%) had some college education. A majority (44/61, 72%) had type 2 diabetes, 59% (36/61) used insulin, and 56% (34/61) reported having current or prior experience with using a CGM. Respondents had overall high levels of general health literacy (mean 13.1, SD 2.6; possible scores: 3-15) and digital health literacy (mean 10.6, SD 2.1; possible scores: 0-12). The characteristics of the interview participants are presented in Table 2.

**Table 1:** Survey respondent characteristics.

Characteristics		Values
**Age group (y; n=61), n (%)**		
	18-44	32 (52)
	45-70	29 (48)
Female sex (n=61), n (%)		23 (38)
**Race and ethnicity (n=61), n (%)**		
	Asian	4 (7)
	Black or African American, non-Hispanic	21 (34)
	White, non-Hispanic	35 (57)
	Multiple	1 (2)
**Education (n=61), n (%)**		
	High school graduate or GED^a^	11 (18)
	Some college	23 (38)
	College graduate or higher	27 (44)
**Time since diabetes diagnosis (y; n=61), n (%)**		
	≤1	3 (5)
	1-3	11 (18)
	3-5	10 (16)
	>5	37 (61)
**Diabetes type (n=59), n (%)**		
	Type 1	15 (25)
	Type 2	44 (75)
**Insulin frequency (among those who used insulin; n=36), n (%)**		
	Once daily	7 (19)
	Twice daily	7 (19)
	≥3 injections daily	15 (42)
	Insulin pump	7 (19)
Take noninsulin diabetes medications (n=61), n (%)		43 (70)
**CGM^b^ use (n=56), n (%)**		
	Currently	32 (57)
	Previously	2 (4)
	Never	22 (39)
**Brief Health Literacy Screen (possible scores: 3-15; n=60)**		
	Score, mean (SD; range)	13.1 (2.6; 6-15)
	Inadequate health literacy (score: ≤9), n (%)	6 (10)
	Adequate health literacy (score: ≥10), n (%)	54 (90)
**Digital Health Care Literacy Scale (possible scores: 0-12; n=61)**		
	Score, mean (SD; range)	10.6 (2.1; 2-12)
	Marginal digital health literacy (score: ≤9), n (%)	11 (18)
	Adequate digital health literacy (score: ≥10), n (%)	50 (82)

^a^GED: General Educational Development Test.

^b^CGM: continuous glucose monitor.

**Table 2:** Interview participant characteristics.

Characteristics		Values
**Age group (y; n=18), n (%)**		
	18-44	6 (33)
	45-70	12 (67)
Female sex, n (%)		9 (50)
**Race and ethnicity (n=18), n (%)**		
	Asian	2 (11)
	Black or African American, non-Hispanic	7 (39)
	White, non-Hispanic	9 (50)
	Multiple	0 (0)
**Education (n=18), n (%)**		
	High school graduate or GED^a^	4 (22)
	Some college	7 (39)
	College graduate or higher	7 (39)
**Time since diabetes diagnosis (y; n=18), n (%)**		
	≤1	1 (6)
	1-3	2 (11)
	3-5	0 (0)
	>5	15 (83)
**Diabetes type (n=18), n (%)**		
	Type 1	2 (11)
	Type 2	16 (89)
**Insulin frequency (among those who used insulin; n=11), n (%)**		
	Once daily	5 (45)
	Twice daily	1 (9)
	≥3 injections daily	5 (45)
	Insulin pump	0 (0)
Take non–insulin diabetes medications (n=18), n (%)		16 (89)
**CGM^b^ use (n=13), n (%)**		
	Currently	9 (69)
	Previously	0 (0)
	Never	4 (31)
**Brief Health Literacy Screen (possible scores: 3-15; n=18)**		
	Score, mean (SD; range)	12.0 (2.5; 7-15)
	Inadequate health literacy (score: ≤9), n (%)	2 (11)
	Adequate health literacy (score: ≥10), n (%)	16 (89)
**Digital Health Care Literacy Scale (possible scores: 0-12; n=18)**		
	Score, mean (SD; range)	10.6 (2.5; 2-12)
	Marginal digital health literacy (score: ≤9), n (%)	4 (22)
	Adequate digital health literacy (score: ≥10), n (%)	14 (78)

^a^GED: General Educational Development Test.

^b^CGM: continuous glucose monitor.

As shown in Table 3, of 53 survey respondents, 43 (87%) reported currently or previously using health apps in 1 of 6 categories or wearable activity trackers. There was variability in completing survey items on specific types of apps or wearable activity trackers (combined app and wearable activity tracker use: n=53; wearable activity tracker use: n=60). Of the 6 types of health apps plus wearable activity trackers surveyed, on average, 2.6 (SD 2.0; range 0-7) types were used. Nearly half (25/53, 47%) of the respondents used ≥3 types of apps and wearable activity trackers. The most frequently used apps were those related to blood glucose level monitoring (37/61, 61%), followed by those related to food (23/61, 38%) and weight (18/61, 30%). Nearly three-fifths (36/61, 59%) of the respondents reported using a wearable activity tracker, including 28% (17/61) who used an exercise app. A similar percentage of CGM users (20/33, 61%) and nonusers (14/22, 64%) used wearable activity trackers. The use of blood glucose level monitoring apps was more common among CGM users than nonusers (28/34, 82% vs 5/22, 23%; *P*<.001) as well as more common among insulin users than nonusers (27/36, 75% vs 10/25, 40%; *P*=.006). However, non-glucose monitoring app use (39/61, 74%) was similar among CGM users and nonusers (16/28, 57% vs 12/20, 60%) as well as among insulin users and nonusers (20/33, 61% vs 12/20, 60%), but there was a trend toward lower use among those aged 45 to 70 years compared to those aged 18 to 44 years (14/27, 52% vs 18/26, 69%); however, these results were not statistically significant (*P*=.20). Participants also reported sharing self-monitored data with their health care providers at similar rates across age groups (17/32, 53% for those aged 18-44 y vs 16/29, 55% for those aged 45-70 y; *P*=.87). Details of health technology use (current or previous) among interview participants are presented in Table 4.

**Table 3:** Health technology use (current or previous) among survey respondents.

		Respondents, n (%)
**Blood glucose level monitoring apps (n=61)**		
	CGM^a^ or glucometer app	31 (51)
	Another website or app	6 (10)
Food tracking app (n=61)		23 (38)
Weight tracking app (n=61)		18 (30)
Exercise app (n=61)		17 (28)
Stress-related, mindfulness, or meditation app (n=61)		14 (23)
Sleep app (n=61)		13 (21)
Wearable activity tracker (n=61)		36 (59)
**Combined total app types and wearable activity tracker use (n=53)**		
	0	7 (13)
	1	13 (25)
	>1	33 (62)
Have tried to share information from apps with health care provider (n=61)		33 (54)
A health care provider recommended health app or wearable activity tracker (n=49)		18 (37)
“Have you ever completed a telehealth video visit?” (n=54)		42 (78)
“Do you use online resources (websites, search engines) to look up health information?” (n=54)		51 (94)
“I own a personal computer, laptop computer, or tablet” (n=54)		52 (96)

^a^CGM: continuous glucose monitor.

**Table 4:** Health technology use (current or previous) among interview participants.

		Participants, n (%)
**Blood glucose level monitoring apps (n=18)**		
	CGM^a^ or glucometer app	9 (50)
	Another website or app	4 (22)
Food tracking app (n=18)		9 (50)
Weight tracking app (n=18)		6 (33)
Exercise app (n=18)		3 (17)
Stress-related, mindfulness, or meditation app (n=18)		6 (33)
Sleep app (n=18)		6 (33)
Wearable activity tracker (n=18)		11 (61)
**Combined total app types and wearable activity tracker use (n=17)**		
	0	0 (0)
	1	5 (29)
	>1	12 (71)
Have tried to share information from apps with health care provider (n=18)		12 (67)
A health care provider recommended health app or wearable activity tracker (n=10)		4 (40)
“Have you ever completed a telehealth video visit?” (n=11)		8 (73)
“Do you use online resources (websites, search engines) to look up health information?” (n=11)		10 (91)
“I own a personal computer, laptop computer, or tablet” (n=11)		11 (100)

^a^CGM: continuous glucose monitor.

As seen in Table 5, combined wearable activity tracker and app use was higher among those with DHLS scores of ≥10, particularly for those using >1 tracker or app, but this did not reach statistical significance (*P*=.09).

**Table 5:** Digital Health Care Literacy Scale (DHLS) scores and app use among survey respondents.

		Respondents with DHLS scores of ≤9, n (%)	Respondents with DHLS scores of ≥10, n (%)	*P* value
**Total app types used (range 0-6)**		11 (100)	42^a^ (100)	.35^b^
	0	4 (36)	6 (14)	
	1	3 (27)	14 (33)	
	>1	4 (36)	22 (52)	
**Wearable activity tracker use**		11 (100)	49 (100)	.1^c^
	No	7 (64)	17 (35)	
	Yes	4 (36)	32 (65)	
**Combined total app type and wearable activity tracker use (range 0-7)**		11 (100)	42 (100)^a^	.09^b^
	0	3 (27)	4 (10)	
	1	4 (36)	9 (21)	
	>1	4 (36)	29 (69)	

^a^Not all participants completed all app use survey questions; hence, the total number of respondents for total app type and wearable activity tracker use are different.

^b^Fisher exact test.

^c^Fisher exact test for 2 × 2 contingency tables (2-sided).

### Interviews

#### Overview

Of the 61 survey respondents, 18 (30%) were invited to complete semistructured interviews examining how technologies were and could be used in daily diabetes self-management. As shown in Table 2, most interviewees (16/18, 89%) had type 2 diabetes, with 45% (5/11) of the interviewees who used insulin requiring ≥3 injections daily. They had high levels of digital health literacy on the DHLS as well as high levels of overall health literacy on the Brief Health Literacy Screen. Half (9/18, 50%) of the interviewees used a CGM or glucometer app, 67% (12/18) shared data with a health care provider, 73% (11/18) had used telehealth, and 91% (10/11) used web-based resources.

**Figure 1:**
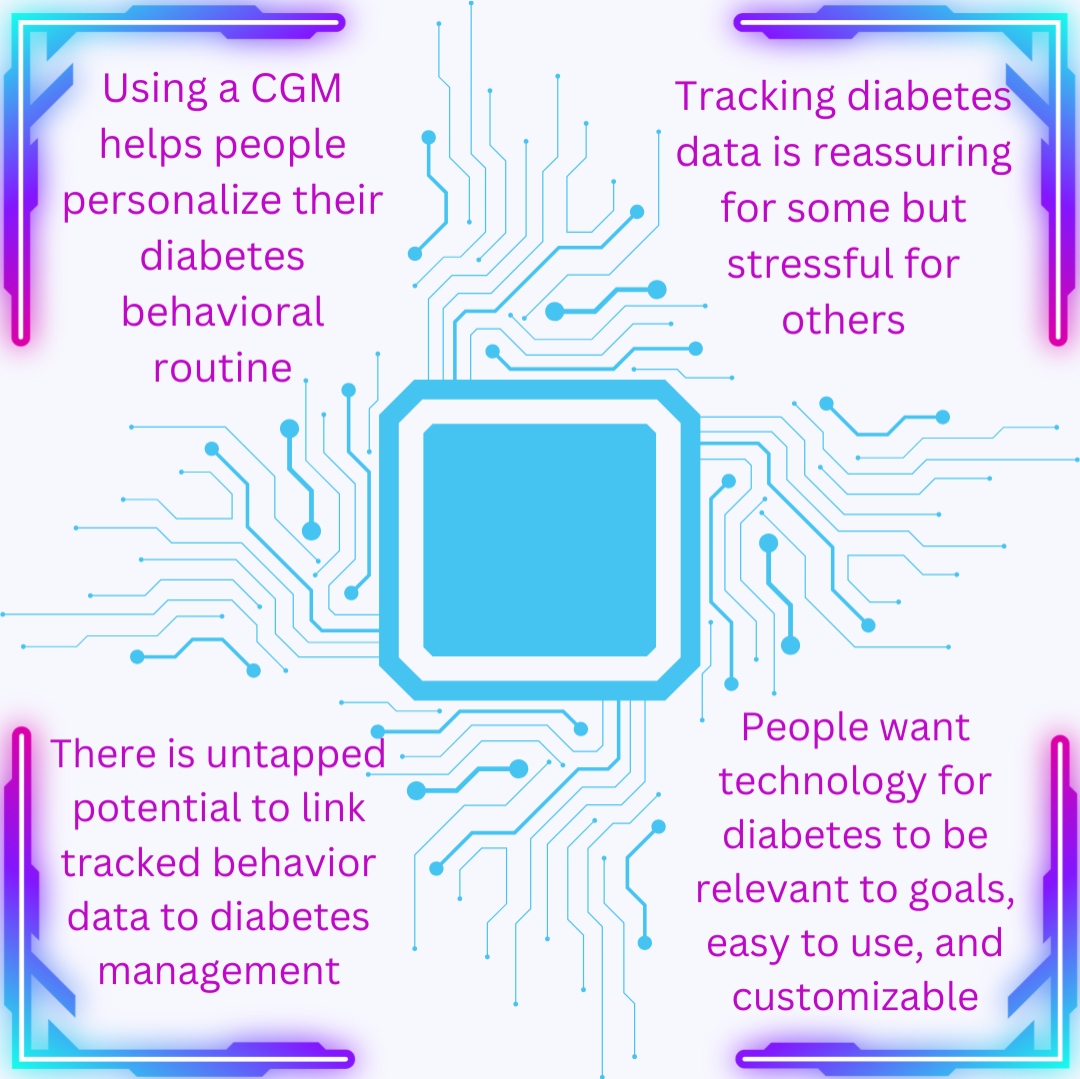
Main themes from semistructured interviews. CGM: continuous glucose monitor.

Four major themes emerged from the interviews, as shown in Figure 1 and described in the following subsections.

#### Theme 1: Using a CGM Helps People Personalize Their Diabetes Self-Management Behavioral Routine

Despite the fact that CGM apps are focused on guiding insulin dose adjustments, many interviewees described using CGM data to guide day-to-day diabetes health behaviors such as eating choices and physical activity patterns:

So I get to choose what to eat knowing what my blood sugar is...When I see my blood sugar’s closer to 200 then I will eat less fruits or sugary food in the morning and really more eggs or something like that.

Male, aged 45-59 y, with type 2 diabetes

Yes, like when I was going to the gym and I was working with this workout group and we were weightlifting, my sugar would go up even though I didn’t eat anything. Like I could start at 90. And by the end of the workout, my sugar was like at 145-150. And I was noticing that happen[ing]...then like on my drive home it was start[ing] to go down but after we worked out with the weights my glucose would always go up.

Female, aged 26-44 y, with type 1 diabetes

Others with type 2 diabetes discussed how the use of their CGM allowed them to be more flexible in terms of when and how often they checked their blood glucose levels:

My doctor absolutely would prefer that I’m, you know, pricking 4 times a day. That’s just not feasible with the lifestyle that I live. It’s just not possible. So the [continuous] glucose monitor helps in that...Sometimes like if I’m feeling weird, I’ll do it [check the app] more times. Sometimes, I’ll do it less times.

Female, aged 26-44 y, with type 2 diabetes

Notably, many participants discussed tailoring the more generic lifestyle behavior advice they receive from clinicians to identify what personally impacts their blood glucose levels. Interviewees described how using CGMs allowed for personalized understanding of the extent to which certain eating patterns impacted their blood glucose levels, which was “better than a dietitian.” They described CGM data as liberating because these data gave them insights into their body’s responses to foods that they previously felt were “off-limits”:

It’s the dietitians I think, are very, to some extent they’re helpful. But I actually found the CGM much more helpful...I like Chinese food. And what I was told was at the beginning, that probably you can’t eat that anymore, in that you have to decrease that. But that’s not entirely true...actually I’m able to actually eat certain types of foods. And I got that information more from my CGM than dietitians.

Male, aged 45-59 y, with type 2 diabetes

It [the CGM] tells me, depending on what I’m going to eat, what I have a taste for, what my taste buds are, yes, no. The numbers will help me and let me know. Okay, I can have this, but not too much of it.

Female, aged 26-44 y, with type 2 diabetes

#### Theme 2: Tracking Additional Data for Diabetes Management Is Reassuring for Some, While Others Feel That It Increases Stress

Interviewees expressed an array of views on how increasing the amount of data available affects their confidence managing diabetes. Some found it reassuring to have extra data:

I told you I was a numbers guy. I’m also kind of a fanatic on schedule, and it was nice, because [the CGM] kind of put you into a schedule.

Male, aged 60-70 y, with type 2 diabetes

Even though the days and the moments I use it [the CGM] fluctuate, I still use it way more than I took the time out to finger stick myself. So even in the days that I’ve only, you know, scanned 3 times. That still gives me a good idea of you know where I stand with my numbers and was able to keep me, you know, mentally aware that, hey? You’re still, you know, you’re still on track. It’s still on track.

Female, aged 26-44 y, with type 2 diabetes

Others who tried tracking health data described feeling stressed or overwhelmed by the additional data:

Each one of us have obsessive compulsive things. And one of the things that bothers me is when I look at the green area in that [CGM app] graph, and I see myself go outside of the green area, it kind of bothers me so I always want to stay within that green area or close to it. I like to see it all green, when I see some yellow I don’t like that. I’ll accept it. And they say yep, that’s because of this food that I ate. But usually I don’t like it.

Male, aged 45-59 y, with type 2 diabetes

So I wait to put my new [CGM on], and getting that first number, I get anxious to see what it is, what it’s going to be. And you know, did I wonder? Like, oh, did I? You know, did I do good today with eating? You know I took my medicine, and you know it should be this, but what if it’s that?

Female, aged 26-44 y, with type 2 diabetes

I think it’s obsessive to be looking at that [activity tracker] all day...I’m not one of those people that wants to count their steps. You know, I might want to count them one day, and then the next day I don’t. So, you know, so I don’t want to be focused on a watch...it’s just too much for me.

Female, aged 49-59 y, with type 2 diabetes

Some people described having extra data from wearable monitors as relieving stress because they were better able to share their data with others:

Every time I scan...my wife gets to see what my blood sugars are. She has the app...So as soon as I scan, it shows up on her phone. It’s one of the alerts in her phone, and then she sees the range as well. So she sees all of the information. So she does remind me and then sometimes she’ll text me and say your sugar’s really high or something and I say “Yeah I just had this type of meal.”

Male, aged 45-59 y, with type 2 diabetes

The ability to share wearable activity tracker and app data with health care providers was also described as stress relieving because interviewees felt that this made it easier for health care providers to understand how diabetes management was going at home:

When I come in for an appointment, [the physician will] download 2 weeks’ worth of data. So she’s connected to my system all the time. And her reaction was, “Wow, you’re 90 plus percent compliant.”

Male, aged 60-70 y, with type 2 diabetes

So the conversations we would have when I go to my appointments...is basically them asking me, okay, what are you doing differently? Your levels are like, really good. There’s no adjustments that need to be made...And then, if they see anything real low on a specific day, they’ll ask me, okay, well, what was going on this day, you were really low. And so there, if there’s any adjustments need to be made, they’ll tell me right then and there.

Female, aged 26-44 y, with type 2 diabetes

#### Theme 3: There Is Untapped Potential to Link Data on Commonly Tracked Lifestyle Behaviors to Diabetes Self-Management

Interviewees mentioned using wearable activity trackers and mobile apps to track multiple aspects of their lifestyle, including healthy eating, physical activity, sleep, and stress levels. While they often discussed links between stress or sleep and blood glucose levels, they rarely discussed linking or comparing tracker data on these behaviors with blood glucose level monitor data:

So I’m one of those people who, you know, who may eat more chips because I’m just feeling down, or I’m just having a stressful day, something like that. And so when that happens when I’m stressed a lot, that’s what messes with my eating, and then it messes with my blood sugar, and then my readings are very high, because I ate the wrong thing all day, or I’ve eaten a wrong...I’ve eaten a candy bar before I went to bed.

Female, aged 45-59 y, with type 2 diabetes and no CGM experience

I do see it [sleep] in my app, my health app, and it shows up that once in a while. That’s a once every four weeks my phone tells me that “oh you reached your goal for tonight.” But it does make me more mindful that yeah, I’m not sleeping as much as I need to be.

Male, aged 45-59 y, with type 2 diabetes

There was a lot of good information [in] there of “Try this or do this, or make sure you’re...” I mean, everything from what you’re eating to socializing. So, I think...what can I, what can I do to sleep better? And how does how does that sleep really affect my diabetes?

Male, aged 60-70 y, with type 2 diabetes discussing a subscription-based lifestyle and weight management app

#### Theme 4: People Prefer to Use Diabetes Management Apps and Wearables When It Is Relevant and Customizable to Their Self-Management Priorities With Data That Are Easily Collected and Integrated in One Place

Interviewees preferred customizing whether, when, and how they tracked certain diabetes lifestyle data based on their personal goals or situation at the time:

It came to a point where I was no longer interested or cared about how many steps I took because, you know, again, most of my day is spent in the car. So like, I wasn’t really stepping. If that makes sense, it wasn’t, you know, you would see different friends and stuff on social media. And, “Oh, I had 10,000 steps,” and it's like, yeah, I barely made a thousand a day. So like, yeah, bump this.

Female, aged 26-44 y, with type 2 diabetes

I think they [specific app] probably try to do too much with exercise logging. So I don’t even, I just ignore that feature. ’Cause doctors really want me to focus on caloric intake.

Male, aged 60-70 y, with type 2 diabetes

I’ll use it [a food and activity tracking app] for myself sometimes to track what I’m eating, and when I was focused on losing weight. And I haven’t really been focused on it because I’m focused on something else right now.

Female, aged 45-59 y, with type 2 diabetes

If they did use trackable data, interviewees wanted to control how frequently they were prompted to track data and what types of prompts they received:

So again, you know I’m technical, technically savvy, whatever you want to call it. And I thought that the app would be perfect for me, thinking, by my lifestyle being on the go and stuff like that. But, it really wasn’t. So one of one of the biggest things that I didn’t like about it, is it overrides. It was overriding anything [settings] that I had on my phone at the time...It was just like, you know, bust through...I didn’t know about the alarms and things so it’s going off, you know, during times where it’s inappropriate.

Female, aged 26-44 y, with type 2 diabetes discussing the mobile app that accompanies the CGM

Interviewees preferred that their data were easy to visualize and interpret:

The application that [the CGM company] provides does provide a graphing capability. So I can graph or print the numbers out for the 3 months time period, and take those along with me for [the physician] to look at...It’ll tell you what your actual numbers were, for the average ones for the day, what the average was for the last 30 days, the last 90 days. And it’ll do a trend line for you. Tell you the time in within range. So all that information is there, and we do share it.

Male, aged 60-70 y, with type 2 diabetes

Yeah. I love [the CGM]. Yeah, I love, It makes things so much easier to put in perspective, like with the graphs and stuff.

Male, aged 45-59 y, with type 2 diabetes

Interviewees felt that the lower the burden of tracking, the better. They expressed a preference for passive collection of data. Manual data entry was viewed as a difficult habit to maintain:

I don't [track] anymore with the [manual entry] activity trackers because they’re more cumbersome than anything, and like I said, that’s why the [watch with activity tracker capability] is working, because it’s just tracking without me being actively needing to work it out. I used the [food and activity tracking app] more for the, for the nutritional information...but then after a month it’s just too cumbersome to log every single thing over there.

Male, aged 45-59 y, with type 2 diabetes

Interviewees preferred seeing data from multiple behaviors in one place, which was described as reducing the burden of data use and, in some cases, helping them make connections between health behaviors and blood glucose levels:

So everything is very integrated in my phone, [the health app] even brings my medications, even brings my labs [and] tests. You know, I look at my sleep and go through the sleep. I look at my steps but because I’m not actively physically active, it’s more of “okay, here’s the information.” It’s nice to see.

Male, aged 45-59 y, with type 2 diabetes

Yes, I have [the CGM] connected to the [CGM] app, and then [other app] is connected to the watch. I just noticed when I was putting in my weight on the [wearable activity tracker] it has the glucose readings on that as well. I guess they’re connecting...it is helpful because they have like the charts. So it’s just nice to see like it’s all in range or it’s going up and down.

Female, aged 26-44 y, with type 1 diabetes

## Discussion

### Principal Findings

In this web-based survey of diverse adults with diabetes and moderate to high digital health literacy, we found that nearly two-thirds (33/53, 62%) used technology to track >1 lifestyle factor impacting their daily diabetes self-management. This included individuals who did not use CGMs and those with varying levels of digital health literacy. It is important to note that participants self-selected to participate in the survey, which was posted on the web, and this may have skewed the sample toward those with higher digital health literacy. Wearable activity trackers were equally used among CGM users and nonusers. Mobile apps used to track blood glucose levels and eating were more common than those used for stress or sleep; however, approximately a quarter of the respondents tracked their stress (13/61, 21%) and sleep (14/61, 23%) levels using apps. In the sample of interviewees with overall higher digital health literacy, we found that current technology offers adults with diabetes an opportunity to customize general diabetes lifestyle advice to their needs and, for some, reduces stress around diabetes management. Given that these adults with diabetes who were able to respond to a web posting to participate in a research study were tracking multiple behaviors, there may be untapped potential, at least among technology-savvy adults with diabetes, to link data from tracking sources to diabetes self-management. Participants desired that apps and wearable activity trackers passively collect, integrate, and graphically display data from various sources in one place and allow customization to their changing personal goals over time.

### Comparison to Prior Work

Our results echo those of prior qualitative studies that identified factors impacting the use of specific individual apps or activity trackers among adults with diabetes. These factors include ease of use, customizable user experiences, health care provider perceptions and guidance, and seamless connectivity. They impacted app and activity tracker use among diverse groups of adults with diabetes (including those on insulin) [26-30]. Our study uniquely focused on how adults with diabetes combined multiple types of diabetes self-management technologies rather than using a particular app or activity tracker. In particular, we were interested in how many people used >1 app because that could present an opportunity to understand how they integrate data from these different sources, particularly because some trackable behaviors (eg, sleep) can impact trackable blood glucose levels or other trackable behaviors (eg, physical activity).

In addition, while some prior studies addressed factors that affect CGM app use [30], our study examined the interface between CGM data, which are more voluminous than those generated by other apps, and diabetes self-management behavior data, which are often tracked on a daily basis. CGM data can be used for overviews, beyond minute-to-minute readings, which could make CGMs and behavior tracking apps easier to use together. There is untapped potential to connect these data sources and, particularly, to help adults with diabetes link data that they are tracking on stress and sleep to their tracked blood glucose levels.

Our results highlight the highly individualized impact of trackable lifestyle data on diabetes self-management behaviors beyond just tracking blood glucose levels. Interviewees who used CGMs described many uses beyond insulin dose adjustments, including using CGMs to guide and personalize their diabetes self-management routines. In this way, additional lifestyle data from novel trackers that cover other domains of diabetes self-management could add more insights and personalization to an individual’s daily diabetes management. In particular, a better understanding of the relationship between personal data patterns and blood glucose levels could increase the sense of ownership of adults with diabetes over their diabetes self-management [31-33] and enhance intrinsic motivation for change [33-35].

### Future Directions

Our study has important implications for how adults with diabetes can use and integrate multiple technologies for diabetes self-management. Participants in our study used apps for tracking multiple behaviors across different age groups and treatment regimens; therefore, technologically focused diabetes self-management education programs could be expanded to accommodate the growing number of non–insulin users who integrate wearable activity tracker, CGM, and app data to manage their condition. These programs could be focused on addressing known barriers among adults with diabetes to using technology for diabetes self-management, including a lack of understanding of how to use personal health data, low digital health literacy, and a lack of knowledge and overall awareness of digital tools used for diabetes [36,37]. Newer platforms that allow users to combine inputs from multiple sources of health data and understand the relationships between these domains have been shown to be acceptable to users [38] and effective in some cases for short-term treatment outcomes in people with type 1 [39,40] and type 2 [41-45] diabetes, although there was heterogeneity in the types of data and interventions included. Taken together, these findings demonstrate the opportunity to incorporate multiple data sources more deliberately in personalized diabetes self-management education and diabetes self-management apps.

### Strengths and Limitations

Our study has several strengths, such as the inclusion of a diverse cohort of adults with diabetes, including people who did not use insulin—especially because more individuals (including those not on insulin) are qualifying for wearable devices such as CGMs and are using health tracking apps.

Our study also has multiple limitations. First, it relies on a convenience sample that is not necessarily representative of the general population of adults with diabetes. In particular, participation was self-selected by people who could use the internet to respond to the study invitation, which could have led to the higher levels of digital health literacy in this sample and may have contributed to higher app and activity tracker use than the general population of adults with diabetes. Second, the experiences of adults with type 1 diabetes and low health literacy are not well represented in the qualitative interview data. Third, because our main goal in this exploratory study was to describe emergent patterns and not quantify associations at this level, we did not collect information about other comorbidities, employment status, geographic location, current blood glucose control, or other factors that might confound technology use. Fourth, the low numbers of participants in the low health literacy and digital health literacy categories limited our power to assess the associations between literacy and technology use. Finally, most of the respondents (58/61, 95%) were aged >25 years; therefore, the results may not be generalizable to teenagers or emerging young adults. The results of this study could guide topics and sampling strategies for future studies that include a wider population-based sample.

### Conclusions

We found that a diverse cohort of adults with diabetes used several wearable and mobile app technologies to track multiple aspects of their daily routines relevant to diabetes self-management. They were interested in digital tools that were easy to use, integrated data across multiple platforms, and aligned with their personal priorities in customizable ways. Our findings have important implications for the ways in which adults with diabetes can be empowered to manage their health successfully and experience the benefits of health technologies.

## Appendix

Multimedia Appendix 1 Key survey items.

Multimedia Appendix 2 Interview guide.

## Abbreviations

CGM: continuous glucose monitor
DHLS: Digital Health Care Literacy Scale

## Data Availability

The datasets generated and analyzed during this study are not publicly available because the small sample size and detailed qualitative responses could make participants identifiable. Requests for deidentified survey data can be sent to the corresponding author and will be considered on a case-by-case basis.
