# Supplementary material for: Examining How Adults With Diabetes Use Technologies to Support Diabetes Self-Management: Mixed Methods Study
Source: JMIR Diabetes. 2025 Mar 25;10:e64505. doi: 10.2196/64505 (PMC11979526; doi:10.2196/64505)
Supplement: Multimedia Appendix 1 [file diabetes_v10i1e64505_app1.docx]

Appendix 1: Key Survey Items

## Part I: Diabetes History

Do you test your own blood sugar levels regularly at home? If yes, what kind of devices do you use?

Continuous glucose monitor (CGM)

Fingerstick device

Both CGM and fingerstick device

Other

Have you ever used a Continuous glucose monitor (CGM)?

Yes, currently use a CGM

Yes, have used a CGM in the past

No, have never used a CGM

Unsure

How long has it been since you first used a CGM at home?

Less than 6 months

6 months – 1 year

1 – 2 years

3 years or more

Unsure

N/A (Not CGM user)

Have you used a CGM continuously (without a break of more than a month) since you first tried it

Yes, I have used it almost continuously

No, I have taken a break of more than a month

Unsure

N/A (Not CGM user)

If you use a fingerstick device, how often do you test your blood sugar at home?

3 or more times per day

Once or twice per day

1-4 times per week

Less than once a week

Never

Unsure

## Part II: Digital Health Care Experience

Have you ever used a website or phone app to track your sugars?

Yes, the app that came with my CGM or glucometer

Yes, another website or app ______________ (fill in)

☐ No

☐ Unsure

Have you ever used a website or phone app to track what you eat? (examples include Noom, LoseIt!, Weight Watchers, or MyFitnessPal)

☐ Yes, _____ (fill in)

☐ No

☐ Unsure

Have you ever used a website or phone app to track your weight?

☐ Yes, _____ (fill in)

☐ No

☐ Unsure

Have you ever used a wearable device to track your walking or step count?

If needed: examples include Fitbits, smart watches, phone apps

☐ Yes ____ (fill in)

☐ No

☐ Unsure

Have you ever used applications on your phone to track or guide other types of exercise (such as Apple Fitness, Peloton, MapMyRun, Nike+ Training Club, Fitbit)?

☐ Yes

☐ No

☐ Unsure

Have you ever used an app, phone or computer for mindfulness, meditation, or stress-relief activities?

☐ Yes ______ (fill in)

☐ No

☐ Unsure

Have you ever used an app, phone or computer to track information about your sleep?

☐ Yes ______ (fill in)

☐ No

☐ Unsure

Have you ever tried to share information from apps (CGM app, Fitbit, food, exercise) with your healthcare provider?

☐ Yes

☐ No

☐ Unsure

Has your healthcare team (doctor, nurse, therapist, care manager) ever recommended for you to use a mobile application for your health?

☐ Yes

☐ No

☐ Unsure

If your healthcare team has recommended a mobile application for your health, how did you use this application?

☐ Free text?

Have you every completed a telehealth visit (video visit) with your healthcare team?

☐ Yes

☐ No

☐ Unsure

Do you use online resources (websites, search engines) to look up information related to your health?

☐ Yes

☐ No

☐ Unsure

I own a smartphone.

☐ Yes

☐ No

☐ Unsure

I own a personal computer, laptop computer, or tablet

☐ Yes

☐ No

☐ Unsure

## BRIEF Health Literacy Screening Tool^i^

These next questions are related to your comfort with reading, understanding, and acting upon information related to your health on your own as well as how often you need help understanding information about your health.

How often do you have someone (like a family member, friend, hospital/clinic worker or caregiver) help you read hospital materials?

All of the time (1 pt)

Most of the time (2 pts)

Some of the time (3 pts)

A little of the time (4 pts)

None of the time (5 pts)

How often do you have problems learning about your medical condition because of difficulty understanding written information?

All of the time (1 pts)

Most of the time (2 pts)

Some of the time (3 pts)

A little of the time (4 pts)

None of the time (5 pts)

How confident are you filling out medical forms by yourself?

All of the time (5 pts)

Most of the time (4 pts)

Some of the time (3 pts)

A little of the time (2 pts)

None of the time (1 pt)

Digital Health Care Literacy Scale^ii^

These next questions ask about your comfort and experience with using digital healthcare – specifically how you are able to use electronic resources and figure out issues with these tools on your own. Please rate your agreement with the following statements ranging from “Strongly Disagree” to “Strongly Agree.”

I can use applications/programs (like Zoom) on my cell phone, computer, or another electronic device on my own (without asking for help from someone else).

☐ Strongly disagree (0 points)

Disagree (1 point)

Neutral (2 points)

Agree (3 points)

Strongly agree (4 points)

I can set up a video chat using my cell phone, computer, or another electronic device on my own (without asking for help from someone else).

☐ Strongly disagree (0 points)

Disagree (1 point)

Neutral (2 points)

Agree (3 points)

Strongly agree (4 points)

I can solve or figure out how to solve basic technical issues on my own (without asking for help from someone else).

☐ Strongly disagree (0 points)

Disagree (1 point)

Neutral (2 points)

Agree (3 points)

Strongly agree (4 points)

## Sources Cited:

1. Chew, Lisa D., Katharine A. Bradley, and Edward J. Boyko. "Brief questions to identify patients with inadequate health literacy." *Family Medicine* 36.8 (2004): 588-594.
2. Nelson, Lyndsay A., et al. "A 3-item measure of digital health care literacy: development and validation study." *JMIR Formative Research* 6.4 (2022): e36043.
