# Supplementary material for: Examining How Adults With Diabetes Use Technologies to Support Diabetes Self-Management: Mixed Methods Study
Source: JMIR Diabetes. 2025 Mar 25;10:e64505. doi: 10.2196/64505 (PMC11979526; doi:10.2196/64505)
Supplement: Multimedia Appendix 2 [file diabetes_v10i1e64505_app2.docx]

Appendix 2: Interview Guide

Part I. Current daily routine for diabetes/current CGM use

| First, I’d like to get a sense of your day to day routine to manage your diabetes. Please walk me through a typical day from the time you wake up. For example, what do you keep track of that helps you manage diabetes? What other times during your day do you think about how your next activity will affect diabetes? | *Prompts*   - If don’t mention tracking sugars, or other habits (eating, activity)   *~Do you track anything related to diabetes?*  *~Why/why not?*  *~If no, have you ever tracked sugars, eating, or activity in the past? Why did you stop?* |
| --- | --- |
| How do you currently manage remembering to check your blood sugar or take medication? | - *(If they use an electronic reminder)* How do you use electronic reminders to do this? - *(If they don’t use an electronic reminder)* How comfortable would you be using an electronic reminder? What would make an electronic reminder most helpful for you? |
| **[CGM users]** Can you describe a time you used your CGM readings or trends to change your behaviors or routine? (*Prompt if needed: change your physical activity, adjust your diet, adjust when you take your medication*)? | - How did you decide to make this change? - Did you get anyone else’s advice? (why/why not) - Did you look up any information how to use your CGM readings to make this kind of change? - How often do you make adjustments like these in response to your CGM readings? |
| **[CGM users]** How often do you show your CGM readings or trends to a healthcare provider (e.g., a doctor, nurse, or diabetes educator)? | - If yes, why did you share your data with your provider? - If yes, can you describe a time you did this, and how the provider used your information?   - Was this helpful to you? Why/why not?   - What would make sharing your CGM data with your healthcare team more helpful? - If no, why not?   - What would make sharing your CGM data with your healthcare team more helpful? |
| **[CGM users]** Describe what it was like to get started using a CGM. For example, what steps did you take to set it up, and did anyone help you? | - What would have made it easier to get started using your CGM/activity monitor information? - How could your doctor/doctor’s office have made this easier/ helped more? - What other types of people or training may have been helpful in getting started with CGM? |
| What monitors or devices **[OTHER THAN CGM]** have been the most helpful to you in terms of figuring out how to manage diabetes from day to day? Please walk me through how you use this during a typical day | - - *Why is using this monitor/device this way helpful?*   - *How did you decide on this strategy?*   - *What was the hardest part about getting started with this?*   - *How do you think this routine affects your stress levels day to day?*   - *What would make this more helpful?* - *[Prompt to describe further if mention ways that their method changed behavior*   *or what kinds of behaviors they think are related to blood sugar]* |

Part II. Experiences Using and Setting Up Diabetes Technology

| **[NON CGM users only]** When you used a [fingerstick glucometer] app or computer program to track your blood sugar levels…. | - Can you describe how you used the information in this app/program to make changes in your daily routine? - What features of this method made it easy to use to decide how to manage diabetes day to day? - What features of this method not useful to decide how to manage diabetes day to day? - Why did you choose this app/program? Did someone recommend it to you? - What would have made it easier to get started using this app/program? - Did you share your sugar data with your healthcare provider or diabetes educator?   - If yes, was it helpful to you? Why/ why not?   - If no, why not? |
| --- | --- |
| **[If they used activity monitor]** When you used an activity monitor to monitor step count or physical activity… | - Can you describe how you use/used the activity monitor information to help manage your blood sugar levels? - Do you feel this monitor helped you better manage diabetes? Why/why not?   - What would have made it more helpful in managing diabetes? - *Why did you choose this app/program? Did someone recommend it to you?* - *What would have made it easier to get started using this app/program?* |
| **Skip for current CGM users** | |
| When you used a guided exercise application (like Nike Training Club or MapMyRun)… | - Can you describe how you use/used the activity monitor information to help manage your blood sugar levels? - Do you feel this monitor helped you better manage diabetes? Why/why not?   - What would have made it more helpful in managing diabetes? - *Why did you choose this app/program? Did someone recommend it to you?* - *What would have made it easier to get started using this app/program?* |
| When you used an app or scale connected to the internet or your phone to monitor your weight… |  |
| When you used an app or computer program to monitor what you ate… |  |
| When you used an app, phone or computer to track sleep…. |  |
| When you used an app, phone or computer for mindfulness, meditation, or to lower stress … |  |
| Would you want to give your healthcare team access to any of your tracking data OTHER than your sugar levels? For example: step counts, scale weights, or food tracking? | - If yes, why? Describe what you think this process should look like. - If no, describe why you wouldn’t want HCP to see your data. - Would it be helpful to share this kind of data with anyone other than a HCP? Who? Why/why not?   - Can prompt them to describe more if they say they’ve done this. How was it/wasn’t it helpful? |
